# Prevalence of and factors associated with suboptimal glycemic control among patients with type 2 diabetes mellitus attending public hospitals in the Greater Male’ Region, Maldives: a hospital-based cross-sectional study

**DOI:** 10.1186/s12889-024-18693-6

**Published:** 2024-04-25

**Authors:** Jeehana Shareef, Tawatchai Apidechkul, Peeradone Srichan

**Affiliations:** 1 School of Health Science, Mae Fah Luang University, Chiang Rai, Thailand; 2 Center of Excellence for the Hill tribe Health Research, Mae Fah Luang University, Chiang Rai, 57100 Thailand

**Keywords:** Diabetes mellitus, Suboptimal glycemic control, Factor associated, Maldives

## Abstract

**Background:**

Suboptimal glycemic control of type 2 diabetes mellitus (T2DM) which is defined as having HbA1c greater than 7% is a major public health problem in several countries, including the Maldives. The study aimed to estimate the prevalence and determine factors associated with suboptimal glycemic control among T2DM patients.

**Methods:**

A hospital-based cross-sectional was applied to collect data from T2DM patients who attended public hospitals in the Greater Male’ Region, Maldives where were one of the highest reports of T2DM and suboptimal glycemic control cases in the country between January to March 2023 by a validated questionnaire and anthropometric measurements. Five (5) ml blood specimens were collected to measure the glycated hemoglobin (HbA1c) level. Univariable and multivariable logistic regressions were employed to determine factors associated with suboptimal glycemic control of T2DM at a significant level of α = 0.05.

**Results:**

A total of 341 participants were recruited for the study: 65.7% were female, 42.5% were aged 40–60 years, and 42.2% were married. The overall prevalence of suboptimal glycemic control was 50.7%. Ten variables were found to be associated with suboptimal glycemic control in multivariable logistic regression. Those aged 40–60 years (AOR = 3.35, 95% CI = 1.78–6.30), being single (AOR = 2.53, 95% CI = 1.21–5.30), preparation of food using more than three tablespoons of cooking oil (AOR = 2.78, 95% CI = 1.46–5.28), preparation of food with more than three tablespoons of sugar (AOR = 2.55, 95% CI = 1.31–4.93), no exercise (AOR = 2.04, 95% CI = 1.15–3.61), DM diagnosed with more than twenty years prior (AOR = 2.59, 95% CI = 1.34–4.99), obese body mass index (BMI) (AOR = 3.82, 95% CI = 1.75–8.32), high total cholesterol (AOR = 2.43, 95% CI = 1.36–4.35), high triglycerides (AOR = 3.43, 95% CI = 1.93–6.11), and high-level stress (AOR = 2.97, 95% CI = 1.48–5.93) were having a greater odds of having suboptimal glycemic control than those who did not have these characteristics.

**Conclusion:**

A large proportion of T2DM patients in the Greater Male’ Region fail to control their blood glucose. Effective public health interventions should be introduced, especially interventions focused on reducing cooking oil and sugar in daily cooking practices, encouraging regular exercise, and maintaining cholesterol levels, particularly for those diagnosed with diabetes mellitus for more than 20 years prior.

**Supplementary Information:**

The online version contains supplementary material available at 10.1186/s12889-024-18693-6.

## Introduction

Diabetes mellitus (DM) is a serious public health concern in low and middle-income countries, especially in type 2 diabetes mellitus (T2DM) patients with suboptimal glycemic control [1], which is defined as HbA1c ≥ 7% [2]. According to the World Health Organization (WHO), T2DM affected 422 million people worldwide, causing 1.5 million deaths annually [3], and an extra 3 million T2DM deaths occurred due to suboptimal glycemic control in 2019 [1]. Aside from T2DM deaths, uncontrolled long-term hyperglycemia can result in the development of macrovascular and microvascular complications, like diabetic nephropathy, neuropathy, cardiovascular disease, and lower limb amputation [1]. It also significantly burdens public health and socioeconomic development in all countries, which requires substantial financial resources for treatment and care, particularly in low-income countries where screening, diagnosis, and treatment are limited [4–7], including the Maldives. Its complications can diminish patients and their family’s quality of life [4, 5].

In the same vein, the country is experiencing a rapid increase in non-communicable diseases (NCDs) due to economic development and globalization. The Maldivians have transitioned from an active lifestyle to a sedentary one, consuming processed foods, high-calorie foods, saturated fats, and sugar. Additionally, most of the Maldivian islands are small and many residents depend on motorbikes rather than walking or cycling, particularly in the Greater Male’ Region. This has increased NCDs, and the primary cause of morbidity and mortality in the country is accounting for up to 81.0% of all deaths [8]. Poor dietary habits, a higher body mass index (BMI), and elevated blood pressure were found to be the top five risk factors for T2DM with other NCD burdens in the country [9]. The availability and promotion of unhealthy foods increase T2DM prevalence [10], with a 6.7% prevalence among people aged 20–79 years [11]. In 2020, 23 DM deaths were attributed in the country, and the disease ranked as the sixth leading cause of NCD-related deaths [12]. Many T2DM patients are diagnosed and treated, but their ability to control their blood sugar level is often very poor [13].

Year by year, the number of T2DM cases is increasing in the country. According to the National Diabetes Center (NDC) at Indira Gandhi Memorial Hospital (IGMH), a total of 1,733 T2DM patients were registered in the NDC between October 2020 and December 2022, with 293 suboptimal glycemic control cases were attended in January 2023 [14]. This was an alarming figure for being a small, populated country. Since there is little or no information regarding suboptimal glycemic control among T2DM patients. Therefore, the study aimed to estimate the prevalence and to determine factors associated with suboptimal glycemic control among T2DM patients attending public hospitals in the Greater Male’ Region, Maldives.

## Methods

### Study design and study setting

A hospital-based cross-sectional study was employed to collect data from T2DM patients attending public hospitals in the Greater Male’ Region of the Maldives, including IGMH, Hulhumale’ Hospital, Vilimale’ Hospital, and Senahiya Hospital.

### Study population and eligible population

Patients with T2DM who attended four selected public hospitals were enrolled in the study population. Those who had been diagnosed with T2DM for at least two years and who attended the IGMH National Diabetic Centre, as well as T2DM patients who attended other public hospitals (Hulhumale Hospital, Villimale Hospital, and Senahiya Hospital) between 24th January and 18th March 2023, met the inclusion criteria. However, those unable to provide the necessary information and pregnant women were excluded from the study.

### Sample size

The sample size of this study was determined using the standard formula for a cross-sectional design [15]; n = [Z^2^_α/2_P (1-P)] / d^2^, where the Z = value from the standard normal distribution corresponded to the desired confidence level (Z = 1.96 for 95% CI), *P* = the expected true proportion (*P* = 0.72) [16], and d = precision (d = 0.05); after adding 10% for non-response and any other error in the study, 341 participants were used as the final sample size for analysis.

### Research instruments

The researcher developed a questionnaire that was used to collect data. It entailed four parts. Part I, eight questions were used to collect sociodemographic information, such as age, gender, level of education, marital status, occupation, monthly income, and living status. Part II, twenty-four questions were used to collect information regarding diabetic self-care factors, such as self-monitoring blood glucose, regular follow-up visits, cigarette smoking, alcohol consumption, food consumption behavior, adherence to exercise, and knowledge about DM prevention and care. In this section, under knowledge about DM prevention and care, ten questions were asked to assess the three levels of knowledge. Part III, consisted of seven clinically relevant questions, i.e., duration of diabetes, hypertension, family history of DM, BMI or obesity, waist-to-hip ratio, lipid profile levels, and stress. The anthropometric measurements (such as height, weight, BMI, waist, and hip circumference), blood pressure, as well as a lipid profile were collected in this part A three mL blood sample was drawn to determine HbA1c level, and a five mL blood sample for lipid profile tests, including total cholesterol, triglycerides, HDL-C, and LDL-C. Under stress, five questions from the stress test (ST-5) [17], were used to determine the stress level. In the last part, questions included the types of antidiabetic medication taken as well as the behavior of taking medication.

### Validated questionnaire

The validity and reliability of the questionnaire were assessed using various methods. The item-objective congruence (IOC) technique [18] was used to assess the validity of the questionnaire. Using this method, three experts evaluated the congruence between each question in terms of how well those questions reflect the content and objectives of the study. Each expert provided a score for each item: “1” means that the question is relevant to the content and objectives of the study, which means that the question does not require any improvement. “0” means that the question is relevant to the content and objectives of the study but needs to be improved before use in the study. “-1” means that the question does not reflect the content and objectives of the study and requires improvement before use. Before interpretation, the average scores were calculated by adding and dividing the scores of three experts. If the average score for the question was less than 0.5, it was eliminated from the final questionnaire. If the questions with scores between 0.5 and 0.7 were to be included in the final questionnaire, they needed to be improved before use. Questions with a score of 0.70 or above were considered for inclusion in the final questionnaire.

Before using the questionnaire in the field, all the questions were tested for reliability with 30 people whose characteristics were similar to those of the study subjects. The pilot testing was conducted on T2DM patients who attended the NDC at IGMH in Male’, Maldives. During this process, the feasibility, proper words or sentences, and order of the questions were assessed. The questionnaire’s knowledge-related questions were subjected to a reliability test (Cronbach’s alpha) with a result of 0.86.

### Measures

Body mass index (BMI) is classified based on the WHO Asian BMI classification standard. BMI is calculated using the formula: a person’s weight in kilograms divided by a height in meters squared (kg/m^2^). It is divided into five categories: underweight (<18.5 kg/m^2^), normal weight (18.5–22.9 kg/m^2^), overweight (23.3–24.9 kg/m^2^), obese 1 (25–29.9 kg/m^2^), and obese 11 (≥ 30 kg/m^2^) [19]. A waist-to-hip ratio (WHR) ≥ 94.0 centimeters for males and ≥ 80.0 centimeters for females was classified as an unhealthy waist-to-hip ratio [20]. WHR was calculated as waist circumference in centimeters divided by hip circumference in centimeters. Patients with a systolic blood pressure of ≥ 140 mmHg and/or diastolic blood pressure of ≥ 90 mmHg, or who use antihypertensive medication regardless of their current blood pressure, were classified as hypertensive [21].

To measure the level of stress among participants, the ST-5 was used, and stress was classified into three categories: low (≤4 scores), moderate (5–7 scores), and high (≥8 scores) [17]. Glycated hemoglobin (HbA1c) levels were classified according to the American Diabetes Association standard, with an HbA1c level of > 7.0% defining suboptimal glycemic control [2] Abnormal lipid profiles were defined as total cholesterol (TC) levels > 200.0 mg/dL [22]. High-density lipoprotein cholesterol (HDL-C) was divided into two main groups based on WHO recommendations: low (<40.0 mg/dL) and normal (≥ 40.0 mg/dL) [20], HDL-C abnormal levels for men (< 40.0 mg/dL) and women (< 50.0 mg/dL) [20]. Low-density lipoprotein cholesterol was classified by WHO recommendations. They are classified into two main groups: normal (<100.0 mg/dL) and high (≥ 130.0 mg/dL) [20]. The triglyceride levels were divided into two groups, which were based on WHO guidelines, which included: optimal (<150 mg/dL) and high (≥ 150 mg/dL) [22].

### Data gathering procedures

The IGMH and the other three designated public hospital directors and chiefs were contacted, an appointment was made, and a brief meeting was held to explain the research objectives and data collection procedures. All T2DM patients who attended the clinic on the day of data collection were invited to participate in the study voluntarily. Those who agreed to participate the study were informed about the study objectives, the data collection and blood sample-taking procedures. Before the beginning of the study, participants were informed and asked to sign a written consent form. Then, the participant’s height, weight, and waist circumference were measured as part of the physical examination. Blood pressure was assessed using the Omron Automatic Inflation Blood Pressure Monitor. Qualified and experienced nurses assessed anthropometric measurements (such as height, weight, and waist circumference) and blood pressure. Next, participants were asked to fill out the questionnaire or self-administered to complete the questionnaire. Participants who could not sign the written consent form were asked to use their fingerprints. The researcher helped them complete the questionnaire for those who couldn’t fill it out by themselves. As part of gathering information from the study subjects, blood specimens were collected from those who hadn’t done the recommended blood tests one week before the date of data collection. In these blood specimen tests, patients were asked to fast (nothing to eat or drink) for at least 12 h to determine clinical laboratory tests such as HbA1c, and lipid profiles. Blood samples were obtained after fasting was completed. A medical technician who has a valid license drew blood samples from each participant. All blood specimens were sent to the same hospital medical laboratory on the same day for analysis.

### Statistical analysis

The data were entered into an Excel sheet, coded, cleaned, managed, and then exported into the SPSS IBM SPSS Statistics software, version 20.0 (SPSS, Chicago, IL) for analysis. Descriptive statistics were used to describe the general characteristics of the participants. While percentages were used to describe categorical data, continuous data were described using the mean and standard deviation (SD) for a normal distribution and the median and interquartile range (IQR) for a skewed distribution. The Chi-square was used to determine whether there was any statistically significant association between independent variables and the outcome variable. Logistic regression was applied to find the risk factors for suboptimal glycemic control at a significance level of a = 0.05. The “stepwise method” method was used as a selection variable in the model. In all phases, the Cox-Snell R^2^, Nagelkerke R^2^, and Hosmer-Lemshow were employed to assess the fit of the model. The variables shown to be significant in the univariable logistic model must be included in the multivariable model. The final estimation models were interpreted after fitting all significant variables in the model.

## Results

### General characteristics of the participants

A total of 341 T2DM patients were enrolled from 4 public hospitals: 200 T2DM cases (58.7%) from Indira Gandhi Memorial Hospital, 56 cases (16.4%) from Hulhumale’ Hospital, 55 cases (16.1%) from Villimale Hospital, and 30 T2DM cases (8.8%) from Senahiya Hospital. Of these, 173 had suboptimal glycemic control (50.7%), and 168 had controlled blood glucose (49.3%).

More than half of the participants, 65.7% were female, 42.5% were aged 40–60 years, 44.3% had attained informal education, and 42.2% were married. Nearly half (49.6%) were unemployed, 50.2% did not receive a monthly income, and 26.4% received less than 10,000 MVR per month. Slightly more than one-third (44.0%) had more than five members in their family, and 47.8% stayed with a spouse. In the alcohol use concern, 341 (100.00%) participants (Table 1).

Four (4) variables were detected in general characteristics, with statistically significant differences between suboptimal glycemic control group and controlled blood glucose groups: age (*p*-value = 0.016), education (*p*-value = 0.002), marital status (*p*-value = 0.031), and occupation (*p*-value = 0.031) (Table 1).

### Prevalence of suboptimal glycemic control

The overall prevalence of suboptimal glycemic control was 50.7% (50.5% in females and 51.3% in males). The age group 40–60 had the highest prevalence of suboptimal glycemic control (58.6%) (Table 1).

**Table 1:** General characteristics of participants

Characteristics	Total		Suboptimal glycemic control				χ^2^	*p*-value
			Yes		No			
	*n*	%	*n*	%	*n*	%		
Total	341	100.0	173	50.7	168	49.3	N/A	A/A
**Sex**								
Female	224	65.7	113	50.5	111	49.6	0.02	0.884
Male	117	34.3	60	51.3	57	48.7		
**Age** (years)								
≤40	137	40.2	57	41.6	80	58.4	8.25	0.016*
40–60	145	42.5	85	58.6	60	41.4		
≥60	59	17.30	31	52.5	28	47.5		
*Min = 25, Max = 79, Mean = 55., and SD = 11.5*								
**Education**								
No schooling	151	44.3	77	51.0	74	49.0	15.34	0.002*
Primary school	92	27.0	33	35.9	59	64.1		
Secondary school	50	14.7	32	64.0	18	36.0		
Tertiary school	48	14.1	31	64.6	17	35.4		
**Marital status**								
Single	89	26.1	44	49.4	45	50.6	6.94	0.031*
Married	144	42.2	84	58.3	60	41.7		
Ever married	108	31.7	45	41.7	63	58.3		
**Occupation**								
Unemployed	169	49.6	74	43.8	95	56.2	8.90	0.031*
Government employee	92	27.0	49	53.3	43	46.7		
Private sector employee	61	17.9	40	65.6	21	34.4		
Self-employed	19	5.6	10	52.6	9	47.4		
**Monthly income** (MVR)								
No income	171	50.2	77	45.0	94	55.0	4.65	0.199
≤ 10,000	90	26.4	52	57.8	38	42.2		
10,001–20,000	61	17.9	34	55.7	27	44.3		
≥ 20,001	19	5.6	10	52.6	9	47.4		
*Min = 1,000, Max = 50,000, Median = 10,000, and IQR = 7,000*								
**Family members**								
Alone	89	26.1	45	50.6	44	49.4	2.23	0.329
≤ 5	102	29.9	46	45.1	56	54.9		
˃ 5	150	44.0	82	54.7	68	45.3		
**Living with**								
Alone	77	22.6	34	44.2	43	55.8	5.17	0.160
Child	55	16.1	26	47.3	29	52.7		
Relatives	46	13.5	20	43.5	26	56.5		
Spouse	163	47.8	93	57.1	70	42.9		

*Significance level α = 0.05

Three variables were found to be associated with suboptimal glycemic control in the univariable analysis in the dimension of socio-demographic characteristics: age, education, and marital status (Table 2).

However, two variables were found to be associated with suboptimal glycemic control in multivariable logistic regression. Participants aged 40–60 years were 3.35 times (95% CI = 1.78–6.30) greater risk of having suboptimal glycemic control, respectively than those aged below 40 and above 60 years. Unmarried participants had 2.53 times (95% CI = 1.21–5.30) greater odds of getting suboptimal glycemic control than those who were married and ever married (Table 2).

**Table 2:** Identifying socio-demographic factors associated with suboptimal glycemic control among T2DM patients by univariable and multivariable logistic regressions

Factors	Suboptimal glycemic control		Univariable analysis			Multivariable analysis		
	Yes (%)	No (%)	OR	95% CI	*p*-value	AOR	95% CI	*p*-value
Total	173 (50.7)	168 (49.3)	*N*/A	*N*/A	*N*/A	*N*/A	*N*/A	*N*/A
**Sex**								
Female	113 (50.5)	111 (49.6)	0.97	0.62–1.51	0.884			
Male	60 (51.3)	57 (48.7)	1.00					
**Age** (years)								
≤40	57 (41.6)	80 (58.4)	1.00			1.00		
40–60	85 (58.6)	60 (41.4)	1.99	1.24–3.19	0.004*	3.35	1.78–6.30	< 0.001*
≥60	31 (52.5)	28 (47.5)	1.55	0.84–2.87	0.159	1.65	0.71–3.87	0.246
**Education**								
No schooling	77 (51.0)	74 (49.0)	0.57	0.29–1.12	0.102			
Primary school	33 (35.9)	59 (64.1)	0.31	0.15–0.64	0.001*			
Secondary school	32 (64.0)	18 (36.0)	0.98	0.43–2.23	0.952			
Tertiary school	31 (64.6)	17 (35.4)	1.00					
**Marital status**								
Single	44 (49.4)	45 (50.6)	1.37	0.78–2.41	0.276	2.53	1.21–5.30	0.014*
Married	84 (58.3)	60 (41.7)	1.96	1.18–3.25	0.009*	1.82	0.94–3.53	0.077
Ever married	45 (41.7)	63 (58.3)	1.00			1.00		
**Occupation**								
Unemployed	74 (43.8)	95 (56.2)	0.70	0.27–1.81	0.464			
Government employee	49 (53.3)	43 (46.7)	1.03	0.38–2.76	0.960			
Private sector employee	40 (65.6)	21 (34.4)	1.71	0.60–4.87	0.312			
Self-employed	10 (52.6)	9 (47.4)	1.00					
**Monthly income** (MVR)								
No income	77 (45.0)	94 (55.0)	0.74	0.29–1.91	0.529			
≤ 10,000	52 (57.8)	38 (42.2)	1.23	0.46–3.32	0.681			
10,001–20,000	34 (55.7)	27 (44.3)	1.13	0.40–3.18	0.812			
≥ 20,001	10 (52.6)	9 (47.4)	1.00					
**Family members**								
Alone	45 (50.6)	44 (49.4)	0.85	0.50–1.43	0.539			
≤ 5	46 (45.1)	56 (54.9)	0.68	0.41–1.13	0.137			
˃ 5	82 (54.7)	68 (45.3)	1.00					
**Living with**								
Alone	34 (44.2)	43 (55.8)	0.60	0.35–1.03	0.063			
Child	26 (47.3)	29 (52.7)	0.68	0.37–1.25	0.209			
Relatives	20 (43.5)	26 (56.5)	0.58	0.30–1.12	0.105			
Spouse	93 (57.1)	70 (42.9)	1.00					

*Significance level α = 0.05

Twelve variables were found to be associated with suboptimal glycemic control in the univariable analysis in the dimension of self-care: method of blood glucose checking, frequency of checking blood glucose, missed DM appointments, number of cigarettes smoked per day, number of meals had daily, food prepared with cooking oil, food prepared with sugar, coconut milk-prepared food consumed on every week, eating sugary foods daily, drinking tea with sugar, juice, and exercise (Table 3).

Three variables were found to be associated with suboptimal glycemic control in multivariable logistic regression. Those who prepared their favorite dish with more than three tablespoons of cooking oil were 2.78 times (95% CI = 1.46–5.28) more likely to have suboptimal glycemic control than those who used less than three tablespoons. Participants who added more than three tablespoons of sugar to their favorite dish had 2.55 times (95% CI = 1.31–4.93) greater odds of developing suboptimal glycemic control than those who added less than three tablespoons. Those who did not exercise regularly had 2.04 times (95% CI = 1.15–3.61) more likely to have suboptimal glycemic control than those who did (Table 3).

**Table 3:** Identifying diabetic self-care and knowledge toward DM prevention and control that associated with suboptimal glycemic control among T2DM patients by univariable and multivariable logistic regressions

Factors	Suboptimal glycemic control		Univariable analysis			Multivariable analysis		
	Yes (%)	No (%)	OR	95% CI	*p*-value	AOR	95% CI	*p*-value
Total	173 (50.7)	168 (49.3)	*N*/A	*N*/A	*N*/A	*N*/A	*N*/A	*N*/A
**Checking blood glucose**								
Medical staff	106 (46.1)	124 (53.9)	1.00					
Themselves at home	67 (60.4)	44 (39.6)	1.78	1.12–2.82	0.014*			
**Frequency of checking blood glucose**								
Few times	23 (63.9)	13 (36.1)	1.00					
Weekly	48 (70.6)	20 (29.4)	1.36	0.58–3.19	0.486			
Monthly	58 (31.5)	126 (68.5)	0.26	0.12–0.55	< 0.001*			
Everyday	44 (83.0)	9 (17.0)	2.76	1.03–7.42	0.044*			
**Missed DM appointment**								
No	122 (55.7)	97 (44.3)	1.75	1.12–2.74	0.014*			
Yes	51 (41.8)	71 (58.2)	1.00					
**Smoking**								
No	120 (49.4)	123 (50.6)	1.00					
Ever	11 (44.0)	14 (56.0)	0.81	0.35–1.85	0.609			
Yes	42 (57.5)	31 (42.5)	1.39	0.82–2.35	0.223			
***Smoking*** *(year)*								
≤ 20	12 (48.0)	13 (52.0)	1.00					
˃ 20	30 (62.5)	18 (37.5)	1.81	0.68–4.80	0.237			
***No cigarettes smoke*** *(day)*								
≤ 10	17 (44.7)	21 (55.3)	1.00					
˃ 10	25 (71.4)	10 (28.6)	3.09	1.17–8.17	0.023*			
**No of the meal** (times/day)								
≤ 3 meals	55 (64.7)	30 (35.3)	2.14	1.29–3.56	0.003*			
˃ 3 meals	118 (46.1)	138 (53.9)	1.00					
**Preparing food**								
Themselves	163 (50.2)	162 (49.9)	1.00					
Buying	10 (62.5)	6 (37.5)	1.66	0.59–4.66	0.339			
**Food prepared with cooking oil** (tablespoon)								
≤ 1	50 (38.8)	79 (61.2)	1.00			1.00		
1–3	29 (40.9)	42 (59.2)	1.09	0.60–1.97	0.773	0.86	0.42–1.79	0.691
> 3	94 (66.7)	47 (33.3)	3.16	1.92–5.20	< 0.001*	2.78	1.46–5.28	0.002*
**Food prepared with sugar** (tablespoon)								
≤ 1	40 (40.4)	59 (59.6)	1.00			1.00		
1–3	34 (48.6)	36 (51.4)	1.39	0.75–2.58	0.292	1.34	0.59–2.99	0.480
> 3	99 (57.6)	73 (42.4)	2.00	1.21–3.31	0.007*	2.55	1.31–4.93	0.006*
**Coconut milk-prepared food** (day(s)/week)								
≤ 3	59 (43.7)	76 (56.3)	1.00					
˃ 3	114 (55.3)	92 (44.7)	1.60	1.03–2.47	0.036*			
**Eating sugary foods**								
Never	43 (38.1)	70 (62.0)	1.00					
Sometimes	100 (58.1)	72 (41.9)	2.26	1.39–3.68	0.001*			
Regularly	30 (53.6)	26 (46.4)	1.88	0.98–3.59	0.057			
**Drinking tea**								
No	10 (47.6)	11 (52.4)	1.00					
Yes	163 (50.9)	157 (49.1)	1.14	0.47–2.76	0.768			
***Drinking tea with sugar***								
Never	35 (27.6)	92 (72.4)	1.00					
Sometimes	86 (66.2)	44 (33.9)	5.14	3.02–8.75	< 0.001*			
Regularly	42 (66.7)	21 (33.3)	5.26	2.74–10.10	< 0.001*			
**Drinking coffee**								
No	50 (46.3)	58 (53.7)	1.00					
Yes	123 (52.8)	110 (47.2)	1.30	0.82–2.05	0.265			
***Drinking coffee with sugar***								
Never	100 (52.1)	92 (47.9)	1.00					
Sometimes	8 (57.1)	6 (42.9)	1.23	0.41–3.67	0.715			
Regularly	16 (59.3)	11 (40.7)	1.34	0.59–3.03	0.485			
**Drinking juice**								
No	104 (57.5)	77 (42.5)	1.78	1.16–2.74	0.008*			
Yes	69 (43.1)	91 (56.9)	1.00					
***Drinking juice with sugar***								
Never	28 (38.4)	45 (61.6)	1.00					
Sometimes	22 (43.1)	29 (56.9)	1.22	0.59–2.52	0.593			
Regularly	19 (52.8)	17 (47.2)	1.80	0.80–4.02	0.155			
**Fruit intake** (daily)								
No	33 (44.6)	41 (55.4)	0.73	0.44–1.23	0.234			
Yes	140 (52.4)	127 (47.6)	1.00					
**Vegetable intake** (daily)								
No	38 (44.2)	48 (55.8)	0.70	0.43–1.15	0.161			
Yes	135 (52.9)	120 (47.1)	1.00					
**Exercise**								
No	115 (58.4)	82 (41.6)	2.08	1.34–3.22	0.001*	2.04	1.15–3.61	0.014*
Yes	58 (40.3)	86 (59.7)	1.00			1.00		
***No of hours spent doing exercise*** *(minutes/day)*								
˂ 30	59 (17.3)	33 (55.9)	1.36	0.70–2.65	0.364			
> 30	85 (24.9)	41 (48.2)	1.00					
**Knowledge regarding DM prevention and control**								
Low	17 (5.0)	9 (52.9)	1.22	0.46–3.29	0.690			
Moderate	109 (32.0)	61 (56.0)	1.38	0.87–2.20	0.171			
High	215 (63.1)	103 (47.9)	1.00					

*Significance level α = 0.05

Eleven variables were found to be associated with suboptimal glycemic control in the univariable analysis in the dimension of clinical history with biomarkers and DM treatment-related experiences: duration of diabetes, family history of hypertension for the mother, BMI, waist-hip ratio, total cholesterol levels, LDL cholesterol, triglycerides, and stress, the type of diabetes medication taken, forgetting to take diabetes medication (weekly), and forgetting to take diabetes medication monthly (Table 4).

Five variables were found to be associated with suboptimal glycemic control in multivariable logistic regression. Participants diagnosed with DM more than twenty years prior had 2.59 times (95% CI = 1.34–4.99) greater odds of having suboptimal glycemic control, respectively, than those diagnosed with DM less than twenty years. Those with an obese BMI were 3.82 times (95% CI = 1.75–8.32) more likely to have suboptimal glycemic control than those with a normal BMI. Participants with high total cholesterol had 2.43 times (95% CI = 1.36–4.35) more likely to have suboptimal glycemic control than those with normal total cholesterol. Participants with high triglyceride had 3.43 times (95% CI = 1.93–6.11) greater odds of getting suboptimal glycemic control than those with optimal triglycerides, and those who had high-level stress had 2.97 times (95% CI = 1.48–5.93) greater chance of having suboptimal glycemic control than those who had low and moderate levels of stress (Table 4).

**Table 4:** Identifying clinical history with biomarkers and DM treatment-related experiences associated with suboptimal glycemic control among T2DM patients by univariable and multivariable logistic regressions

Factors	Suboptimal glycemic control		Univariable analysis			Multivariable analysis		
	Yes (%)	No (%)	OR	95% CI	*p*-value	AOR	95% CI	*p*-value
Total	173 (50.7)	168 (49.3)	*N*/A	*N*/A	*N*/A	*N*/A	*N*/A	*N*/A
**Length of DM diagnosed** (years)								
≤ 10	47 (45.2)	57 (54.8)	1.00			1.00		
10–20	40 (37.0)	68 (63.0)	0.71	0.41–1.24	0.228	0.89	0.44–1.81	0.756
˃ 20	86 (66.7)	43 (33.3)	2.43	1.43–4.13	0.001*	2.59	1.34–4.99	0.005*
**Having HT**								
No	53 (54.1)	45 (45.9)	1.65	0.67–4.07	0.278			
Yes	110 (50.2)	109 (49.8)	1.41	0.60–3.32	0.427			
Do not know	10 (41.7)	14 (58.3)	1.00					
***Length of HT diagnosed*** *(years)*								
≤ 5	46 (53.5)	40 (46.5)	1.00					
˃ 5	64 (48.1)	69 (51.9)	0.81	0.47–1.39	0.438			
***Taking medication for HT***								
No	72 (51.4)	68 (48.6)	1.00					
Yes	101 (50.3)	100 (49.8)	0.95	0.62–1.47	0.830			
**Having kidney disease**								
No	118 (49.0)	123 (51.0)	0.58	0.32–1.05	0.070			
Yes	20 (45.5)	24 (54.6)	0.50	0.22–1.12	0.091			
Do not know	35 (62.5)	21 (37.5)	1.00					
***Length of kidney disease diagnosed*** *(years)*								
≤ 5	12 (46.2)	14 (53.9)	1.00					
˃ 5	8 (44.4)	10 (55.6)	0.93	0.28–3.12	0.911			
***Taking medication for kidney disease***								
No	153 (51.5)	144 (48.5)	1.00					
Yes	20 (45.5)	24 (54.6)	0.78	0.42–1.48	0.454			
**Family history of DM** (father)								
No	52 (52.0)	48 (48.0)	1.44	0.86–2.43	0.169			
Yes	80 (53.7)	69 (46.3)	1.35	0.76–2.38	0.304			
Do not know	41 (44.6)	51 (55.4)	1.00					
**Family history of DM** (mother)								
No	51 (51.5)	48 (48.5)	1.59	0.93–2.72	0.087			
Yes	86 (54.4)	72 (45.6)	1.42	0.79–2.54	0.243			
Do not know	36 (42.9)	48 (57.1)	1.00					
**Family history of DM** (grandfather)								
No	41 (55.4)	33 (44.6)	0.76	0.38–1.51	0.430			
Yes	17 (43.6)	22 (56.4)	1.22	0.72–2.07	0.458			
Do not know	115 (50.4)	113 (49.6)	1.00					
**Family history of DM** (grandmother)								
No	29 (49.2)	30 (50.9)	1.35	0.72–2.50	0.350			
Yes	28 (57.1)	21 (42.9)	0.98	0.55–1.73	0.931			
Do not know	116 (49.8)	117 (50.2)	1.00					
**Family history of HT** (father)								
No	47 (51.1)	45 (48.9)	1.52	0.91–2.55	0.113			
Yes	85 (54.5)	71 (45.5)	1.33	0.74–2.36	0.341			
Do not know	41 (44.1)	52 (55.9)	1.00					
**Family history of HT** (mother)								
No	73 (49.7)	74 (50.3)	1.94	1.09–3.43	0.024*			
Yes	63 (58.9)	44 (41.1)	1.33	0.78–2.27	0.291			
Do not know	37 (42.5)	50 (57.5)	1.00					
**Family history of HT** (grandfather)								
No	42 (52.5)	38 (47.5)	0.57	0.26–1.26	0.570			
Yes	11 (37.9)	18 (62.1)	1.03	0.62–1.72	0.905			
Do not know	120 (51.7)	112 (48.3)	1.00					
**Family history of HT** (grandmother)								
No	43 (53.8)	37 (46.3)	1.10	0.49–2.43	0.823			
Yes	14 (51.9)	13 (48.2)	1.18	0.71–1.97	0.519			
Do not know	116 (49.6)	118 (50.4)	1.00					
**Systolic blood pressure** (mm/Hg)								
Normal (˂ 140)	112 (52.6)	101 (47.4)	1.00					
High (≥ 140)	61 (47.7)	67 (52.3)	0.82	0.53–1.27	0.379			
**Diastolic blood pressure** (mm/Hg)								
Normal (˂ 90)	153 (49.8)	154 (50.2)	1.00					
High (≥ 90)	20 (58.8)	14 (41.2)	1.44	0.70–2.95	0.322			
**BMI** (kg/m^2^)								
Normal (18.50–22.9)	26 (32.5)	54 (67.5)	1.00			1.00		
Underweight (< 18.50)	26 (44.8)	32 (55.2)	1.69	0.84–3.39	0.142	1.46	0.59–3.60	0.412
Overweight (23- 24.9)	27 (47.4)	30 (52.6)	1.87	0.93–3.76	0.080	1.87	0.77–4.57	0.167
Obese (> 25)	94 (64.4)	52 (35.6)	3.75	2.11–6.69	< 0.001*	3.82	1.75–8.32	0.001*
**Waist-hip ratio (**W/H in cm)								
Healthy	62 (43.7)	80 (56.3)	1.00					
Unhealthy	111 (55.8)	88 (44.2)	1.63	1.06–2.51	0.028*			
**Total cholesterol** (mg/dL)								
Normal (˂ 200)	52 (41.3)	74 (58.7)	1.00			1.00		
High (˃ 200)	121 (56.3)	94 (43.7)	1.83	1.17–2.86	0.008*	2.43	1.36–4.35	0.003*
**LDL cholesterol** (mg/dL)								
Normal (˂ 100)	69 (42.6)	93 (57.4)	1.00					
High (˃ 100)	104 (58.1)	75 (41.9)	1.87	1.22–2.87	0.004*			
**HDL cholesterol** (mg/dL)								
Low (˂ 40)	67 (51.5)	63 (48.5)	1.05	0.68–1.63	0.815			
Normal (≥ 40)	106 (50.2)	105 (49.8)	1.00					
**Triglyceride** (mg/dL)								
Optimal (˂ 150)	69 (41.6)	97 (58.4)	1.00			1.00		
High (≥ 150)	104 (59.4)	71 (40.6)	2.06	1.34–3.17	0.001*	3.43	1.93–6.11	< 0.001*
**Stress test** (ST-5)								
Low	33 (33.0)	67 (67.0)	1.00			1.00		
Moderate	47 (48.5)	50 (51.6)	1.91	1.07–3.40	0.028*	1.51	0.72–3.17	0.281
High	93 (64.6)	51 (35.4)	3.70	2.16–6.35	< 0.001*	2.97	1.48–5.93	0.002*
**Type of medicine taken for DM**								
OHA	140 (50.7)	136 (49.3)	1.00					
OHA + insulin	16 (38.1)	26 (61.9)	0.60	0.31–1.16	0.130			
Insulin	17 (73.9)	6 (26.1)	2.75	1.05–7.19	0.039*			
**Forgot to take DM medication** (day(s)/week)								
No	123 (45.22)	149 (54.8)	1.00					
≤ 3	40 (76.92)	12 (23.1)	4.04	2.03–8.03	< 0.001*			
˃ 3	10 (58.82)	7 (41.2)	1.73	0.64–4.68	0.280			
**Forgot to take DM medication** (month)								
No	112 (46.86)	127 (53.1)	1.00					
Yes	61 (59.80)	41 (40.2)	1.69	1.05–2.70	0.029*			
**Having side effects from DM drug**								
No	154 (51.9)	143 (48.2)	1.40	0.60–3.29	0.441			
Yes	9 (42.9)	12 (57.1)	0.98	0.30–3.22	0.967			
Do not know	10 (43.5)	13 (56.5)	1.00					
**Medical expenses**								
Covered by Aasandha	157 (53.4)	137 (46.6)	2.29	0.77–6.87	0.139			
Private health scheme	11 (34.4)	21 (65.6)	1.05	0.29–3.84	0.944			
Self-paid	5 (33.3)	10 (66.7)	1.00					

*Significance level α = 0.05

## Discussion

A large proportion of the T2DM patients in the Greater Male’s Region suffer from suboptimal glycemic control, particularly in older age, females, and single. While most people live with low socioeconomic status. Many cooking practices use high volumes of sugar and cooking oil, which leads to high BMI. Lack of regular exercise and high stress are also detected among T2DM patients, which are associated with suboptimal glycemic control.

The prevalence of suboptimal glycemic control among T2DM patients attending public hospitals was extremely high (50.7%), which was in line with a study conducted in Brazil (47.3%) [5]. However, this proportion is shown to be higher in studies conducted in northern Thailand (54.8%) [23], Ethiopia 71.9% [24], India (64.1%) [25], Bangladesh (71.8%) [16], and Saudi Arabia (75.9%) [26]. These variations could be attributable to rapid urbanization, cultural attitudes and beliefs, behavioral and clinical characteristics, availability of health services, income, lack of uniform guidelines, and a lack of patient awareness regarding diabetes prevention and care.

In this study, age was identified as an associated factor that contributes to suboptimal glycemic control. Participants aged 40–60 years had a greater chance of having suboptimal glycemic control than those in the age groups below 40 and above 60 years. This coincided with a study conducted in Western Ethiopia [27], which reported that T2DM patients between the ages of 41 and 60 were more likely to develop suboptimal glycemic control than those in the age groups below 40 and above 60. However, a study conducted in Eastern Sudan [28] did not detect any association between age and suboptimal glycemic control. Contrary to the findings of this study, a study conducted in Ethiopia [21] revealed that T2DM patients over the age of 50 had a greater risk of having suboptimal glycemic control compared to those below the age of 50. The possible reason for suboptimal glycemic control among people in the Greater Male’ Region could be that this age group is a working age group. They may have a busy daily life, which results in difficulty seeking health care, exercising, or adhering to medical recommendations and makes it difficult to control their blood glucose levels.

Being single was detected as another contributor associated with suboptimal glycemic control in this study. Unmarried participants had greater odds of experiencing suboptimal glycemic control than those who were married and ever married. This is in line with results obtained from studies conducted in Northwestern Nigeria [28], Eastern Sudan [29], and Ethiopia [21], which reported that being unmarried was at greater risk of having suboptimal glycemic control than being married. However, a study conducted in northeast Ethiopia showed no significant association between marital status and glycemic control [30]. In contrast, a study conducted in northern Thailand found that married T2DM patients had greater odds of having suboptimal glycemic control compared to their unmarried counterparts [23]. Perhaps it was assumed that unmarried patients might not receive adequate support from their families in terms of clinic attendance, adherence to a healthy diet, and medication as directed. Maybe this could be the reason they were not achieving glycemic levels. Even though in our study, marriage status was not found to be associated with suboptimal glycemic control, a study in Ethiopia [31] reported that it was a protective factor to the suboptimal glycemic control. Another study [32] conducted in Oman reported that a single marital status was associated with suboptimal glycemic control. It is important to investigate the associations between social determinants and suboptimal glycemic control in any social context for further considering effective public health intervention.

The present study showed that cooking oil beyond the recommended daily was associated with suboptimal glycemic control. Participants who prepared their favorite dish using more than three tablespoons of cooking oil were more likely to have suboptimal glycemic control than those who used less than three tablespoons of cooking oil. Thus, this factor tends to play a significant role in developing suboptimal glycemic control and coronary heart disease. A scoping review reported that people with DM should limit their daily intake of cooking oil to a maximum of three teaspoons to manage their diabetes condition effectively [31]. The possible reason for the suboptimal glycemic control observed among people in the Greater Male’ Region could be that deep-frying oily foods is a more common practice in Maldivian culture, and palm oil is the most common oil used for deep frying.

In the same way, this study also found that using excessive amounts of sugar in daily cooking practices was associated with suboptimal glycemic control. Those who added more than three tablespoons of sugar to their favorite dish had a greater risk of developing suboptimal glycemic control than those who added less than three. This finding was consistent with the study conducted in Eastern Sudan, which reported that adding sugar to beverages increased the risk of poor glycemic control [29]. High added sugar intake lowers the hepatic insulin sensitivity index and increases hepatic lipogenesis and visceral fat, boosting blood insulin levels in DM patients [32]. Furthermore, the Maldivian population has observed an increase in the consumption of sugary foods and drinks with added sugar in recent decades [8]. Traditional Maldivian sweets, drinks, pudding, cakes, pastries, baked foods, and areca nut products contain high-added sugar.

This study detected exercise as a predictor associated with suboptimal glycemic control. Participants who did not exercise regularly had greater odds of having suboptimal glycemic control than those who exercised regularly. This finding was supported by studies conducted in Ethiopia [21], Northeast Nigeria [33], Yemen [34], Uganda [35], and Saudi Arabia [26], which reported that those who were not engaged in physical activity had a greater risk of developing suboptimal glycemic control than those who did exercise. Exercising may lower blood glucose levels because active muscles absorb more glucose than resting muscles, which enhances insulin receptors and sensitivity [36]. One possible reason people in the Greater Male’ Region avoid exercise may be due to a lack of time and always being occupied with daily work to support their family.

The duration of diabetes was identified as a significant positive factor in this study. Those diagnosed with DM for more than twenty years prior had greater odds of developing suboptimal glycemic status compared to those who had been diagnosed with DM for less than twenty years. This result was confirmed by studies in Ethiopia [37], Saudi Arabia [38], northern Thailand [23], Nepal [39], and India [40], which discovered that people who were diagnosed with diabetes more than ten years ago were more likely to have poor glycemic control than those diagnosed with diabetes less than ten years. A prolonged period of T2DM is often accompanied by a gradual reduction of insulin production due to pancreatic β-cell failure, which in turn increases insulin resistance, making it more difficult to manage blood glucose [27]. This could be the reason for T2DM patients frequently having suboptimal glycemic control.

Moreover, participants with an obese BMI were more likely to have suboptimal glycemic control than those with normal BMI. This finding was consistent with findings from studies conducted in Ethiopia [6], Saudi Arabia [38], and India [40], which reported that those with an obese BMI had a greater likelihood of developing suboptimal status compared to those with a normal body weight. Obesity causes an increase in the release of Non-Esterified Fatty Acids from adipose tissue, which has been associated with insulin resistance [36]. This might be a possibility for obese diabetics who have poor glycemic control.

TC was discovered to be an important modifiable risk factor associated with suboptimal glycemic control. Participants with high total cholesterol levels were at greater risk of developing suboptimal glycemic control than those with normal total cholesterol. This finding was similar to the study conducted in Southwest Ethiopia [36], and Oman [41] which reported that high total cholesterol had more likelihood of developing suboptimal glycemic control. A possible justification may be the relationship between glycemic control and its influences on total cholesterol in T2DM patients.

Participants with elevated triglyceride levels had a higher risk of developing suboptimal glycemic status than those with optimal triglyceride levels. This was confirmed by a study conducted in India [40], which revealed that DM patients frequently had lipid problems and dyslipidemia was associated with suboptimal glycemic control, especially those with triglycerides > 150 mg/dL. This might occur due to the persistent fatty acid entry into the β cell, resulting in pancreatic β cell dysfunction, which leads to insulin resistance and makes it difficult to manage blood glucose levels [21].

Finally, the results of this study revealed that stress had a significant association with suboptimal glycemic control. Participants who experienced high stress levels had a greater chance of having suboptimal glycemic control than those who experienced moderate or low stress. A study conducted in Iran [42] showed that stress management reduces HbA1c levels among T2DM patients. It is more common among DM patients and has a dual function in its association with DM, like cause and effect. Stress increases HbA1c, whereas diabetes and its complications increase stress in people with T2DM, particularly physical and emotional stress [42].

Throughout the study, some limitations were identified that may have impacted the analysis and interpretation of the findings. First, the design of this study might not be able to apply to identify the causal relationship between independent variables and suboptimal glycemic control due to assessing both exposures and outcomes at the same time. Second, due to the inability to obtain T2DM statistics from designated hospitals, it was challenging to estimate the sample size for each hospital in this study. Third, some questions asked about participants’ experiences might cause recall bias. Lastly, the study settings were hospitals, then generalizing the findings to the population is limited.

## Conclusions

A large proportion of T2DM patients in the Greater Male’ Region fail to control their blood glucose. Effective public health interventions should be introduced, especially interventions focused on reducing cooking oil and sugar in daily cooking practices, encouraging regular exercise, and maintaining cholesterol levels, particularly for those diagnosed with T2DM for more than 20 years prior. Policymakers at all levels should be informed of the information to create a proper approach for further national policy development and implementation.

### Electronic supplementary material

Below is the link to the electronic supplementary material.

Supplementary Material 1

## Data Availability

Supported data for the study findings are available in supplement files.

## Abbreviations

BMI: Body mass index
DM: Diabetes mellitus
Hemoglobin A1c: HbA1c
IOC: Item objective congruence
NCDs: Noncommunicable diseases
NDC: National Diabetes Center
T2DM: Type 2 diabetes mellitus
WHO: World Health Organization
WHR: Waist hip ratio
