# Supplementary material for: Prevalence of and factors associated with suboptimal glycemic control among patients with type 2 diabetes mellitus attending public hospitals in the Greater Male’ Region, Maldives: a hospital-based cross-sectional study
Source: BMC Public Health. 2024 Apr 25;24:1166. doi: 10.1186/s12889-024-18693-6 (PMC11047027; doi:10.1186/s12889-024-18693-6)
Supplement: Supplementary file 1 — Supplementary Material 1 [file 12889_2024_18693_MOESM1_ESM.pdf]

**NID: --------------------**

# QUESTIONNAIRE

## PREVALENCE AND PREDICTORS OF SUBOPTIMAL GLYCEMIC CONTROL AMONG PATIENTS WITH TYPE 2 DIABETES MELLITUS ATTENDING PUBLIC HOSPITALS IN THE GREATER MALE' REGION, MALDIVES

**Blood glucose** ☐ Controlled ☐ Uncontrolled

**Part 1** General information

1.1 Age……………...……. years

1.2 Sex ☐ Male ☐ Female

1.3 Educational level

☐ Never attended school ☐ Primary school completed

☐ Secondary school completed ☐ Higher secondary school completed

☐ University degree

1.4 Marital status

☐ Single ☐ Married ☐ Ever married

1.5 Occupation

☐ Unemployed ☐ Government employee ☐ Self-employed

☐ Agriculturist ☐ Fisherman ☐ Tourism

☐ Construction ☐ Others (specify)---------------------------------

1.6 Income (per month) .............................................Rufiyaa

1.7 How many members are there in your family?

□ Only you □ 1-4 members □ 5-10 members □ More than 11 members

1.8 Living with □ Parents □ Husband/wife □ Daughter

□ Alone □ Others (specify)……………………...

**Part 2** Diabetic self-care

2.1 How do you check your blood glucose level?

□ Medical staff □ Yourself at home

2.2 How often do you check your blood glucose level?

□ Few times □ Everyday □ Weekly □ Monthly

2.3 How often do you miss appointment for your diabetes problem?

□ Never missed □ Sometimes □ All the time

2.4 Do you smoke cigarette?

□ No □ Ever □ Yes---------------years (If **"Yes"** Please answer the question number 2.5)

2.5 On average, how many cigarettes do you smoke a day? --------------cigarette per day.

2.6 Have you ever consumed an alcoholic drink?

□ No □ Yes------------year (If **"Yes"** Please answer the question number 2.7)

2.7 How frequently have you had alcoholic drink?

□ 1-4 days/week □ 5-6 days/week □ Everyday □Monthly

2.8 How often do you exercise (involving moderate to vigorous activity such as jogging, bicycling, running, or swimming) per week?

☐ Never ☐ 1-3 days/week ☐ 4-6 days/week ☐ Everyday

2.9 How many hours do you exercise a day?

☐ Less than 30 minutes ☐ 30 minutes to 1 hour ☐ Over 1 hour

2.10 How many **meals** do you eat in a typical day? --------------- meals per day

2.11 How did you mainly get daily food? ☐ Self-cooking ☐ Buying

2.12 How much of your favorite food is prepared with cooking oil?

☐ 1 Tablespoon ☐ 2 Tablespoons ☐ More than 3 tablespoons

2.13 How much of your favorite food is prepared with sugar?

☐ 1 Tablespoon ☐ 2 Tablespoons ☐ More than 3 tablespoons

2.14 How often do you eat coconut milk-prepared meals at home?

☐ Few times ☐ 1-3 days/week ☐ 4-6 days/week ☐ Everyday

2.15 How often do you eat sugary foods on a daily basis?

☐ Never eat with sugar ☐ Sometimes ☐ All the time

2.16 Do you drink **tea** on regular basis?

☐ No ☐ Yes (If **"Yes"** Please answer the question number 2.17)

2.17 On a typical day, how often do you drink **tea** with **sugar**?

☐ Never drink with sugar ☐ Sometimes ☐ All the time

2.18 Do you drink **coffee** on regular basis?

☐ No ☐ Yes (If **"Yes"** Please answer the question number 2.19)

2.19 On a typical day, how often do you drink **coffee** with **sugar**?

☐ Never drink with sugar ☐ Sometimes ☐ All the time

2.20 Do you drink **juice** on regular basis?

☐ No ☐ Yes (If **"Yes"** Please answer the question number 2.21)

2.21 On a typical day, how often do you drink **juice** with **sugar**?

☐ Never drink with sugar ☐ Sometimes ☐ All the time

2.22 How much do you eat **fruit** each day?

☐ Never ☐ ¼ of the plate ☐ More than ¼ of the plate

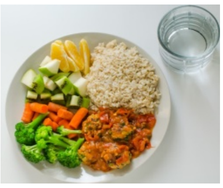

2.23 How much do you eat **vegetables** each day?

☐ Never ☐ ¼ of the plate ☐ More than ¼ of the plate

2.24 Assessment of knowledge about diabetes prevention and control

| Items | True | False | Not sure |
|---|---|---|---|
| 1) If a parent has diabetes, then their children have a chance of developing diabetes. | | | |
| 2) Frequently urinating could be an early sign of diabetes. | | | |
| 3) People with diabetes are more likely to develop blindness | | | |
| 4) Eating too much sugar is not a risk factor for diabetes. | | | |
| 5) Being older than 30 years of age is a risk factor for diabetes. | | | |
| 6) Diabetes is not a risk factor for high blood pressure | | | |

| Items | True | False | Not sure |
|---|---|---|---|
| 7) Being overweight reduces the risk of developing diabetes. | | | |
| 8) Regular physical activity can reduce the risk of developing diabetes. | | | |
| 9) Diet control increases the risk of developing diabetes | | | |
| 10) Diabetes is curable | | | |

**Part 3** Clinical related

3.1 How long have you been diagnosed as diabetes____________ years

3.2 Do you have hypertension?

□ No □ Not sure □ Yes____________ years (If **"Yes"** Please answer the question number 3.3)

3.3 Do you take medication for **hypertension**? □ No □ Yes

3.4 Do you have kidney disease?

□ No □ Not sure □ Yes____________ years (If **"Yes"** Please answer the question number 3.5)

3.5 Do you take medication for **kidney disease**? □ No □ Yes

3.6 Family history of diabetes and hypertension

| History | Diabetes | | | Hypertension | | |
|---|---|---|---|---|---|---|
| | **Yes** | **No** | **Not sure** | **Yes** | **No** | **Not sure** |
| Father | | | | | | |
| Mother | | | | | | |
| Grandfather | | | | | | |
| Grandmother | | | | | | |

3.7 Physical examinations

Systolic blood pressure pressures ………mm/Hg………mm/Hg………mm/Hg

Diastolic blood pressure pressures ………mm/Hg………mm/Hg………mm/Hg

Weight ………...kg

Height ……......cm

Waistline circumference…………….cm

Hip circumference…………………...cm

HbA1c……………………………………mg%

Total cholesterol level ……………………mg/dL

LDL level………………………...mg/dL

HDL level………………………...mg/dL

Triglyceride level ……………………mg/dL

3.8 Stress assessment (ST-5) Please indicate your experience within two weeks

**"No"** means no symptom experience

**"Sometime"** means 1-2 times/week experience symptoms

**"Often"** means 5-6 times/week experience symptoms

**"Regularly"** means 7 times/week experience symptoms

| Feeling | No | Sometime | Often | Regularly |
|---|---|---|---|---|
| 1. Within the last two weeks, did you feel any difficulty sleeping? | | | | |
| 2. Within the last two weeks, did you feel a lack of concentration? | | | | |
| 3. Within the last two weeks, did you feel Irritability? | | | | |
| 4. Within the last two weeks, did you feel bored? | | | | |
| 5. Within the last two weeks, I did not feel like going out and meeting people. | | | | |

**Part 4** Medication Related

4.1 Types of antidiabetic medication taking

☐ Glibenclimide alone ☐ Metformin+Glibenclimide ☐ Insulin alone

☐ Metformin + Insulin ☐ Metformin alone

☐ Others (specify) ……………………………

4.2 How often did you forget to take your diabetes medication in the last week?

☐ Never ☐ 1-3 days/week ☐ 4-6 days/week ☐ Everyday

4.3 Did you forget to take your diabetes medication in the last month?

☐ No ☐ Yes

4.4 Do you have any experiences or side effects from diabetes medication?

 No  Yes  Not sure

**Part 5** Environmental factors

5.1 Who is the main financial supporter of your daily living expenses?

 Parents  Husband/wife  Son  Daughter

 Yourself  Others (specify)………………………….

5.2 Who financially supports and manages your food?

 Parents  Husband/wife  Son  Daughter

 Yourself  Others (specify)………………………….

5.3 Who is normally taking you to the hospital?

 Parents  Husband/wife  Son  Daughter

 Yourself  Others (specify)………………………….

5.4 How do you normally go to the hospital?

 Motorcycle  Car  Bus  By walk (If **"By walk** and **motorcycle**" no need to answer question number 5.5)

5.5 Who is paying for your transport while going to the hospital?

 Parents  Husband/wife  Son  Daughter

 Pay yourself  Others (specify)………………………….

5.6 Is there anyone who is helping you follow medical advice?

 No  Yes

5.7 How do you handle your medical expenses?

 Aasandha  Private insurance  Pay yourself
